# Therapeutic Hypothermia Activates the Endothelin and Nitric Oxide Systems after Cardiac Arrest in a Pig Model of Cardiopulmonary Resuscitation

**DOI:** 10.1371/journal.pone.0064792

**Published:** 2013-05-23

**Authors:** Frank Zoerner, Lars Wiklund, Adriana Miclescu, Cecile Martijn

**Affiliations:** 1 Department of Surgical Sciences/Anesthesiology and Intensive Care, Uppsala University, Uppsala, Sweden; 2 Department of Operative and Intensive Care Medicine, Hallands Hospital Halmstad, Halmstad, Sweden; 3 Science for Life Laboratory, Uppsala, Sweden; 4 Department of Chemistry-BMC, Uppsala University, Uppsala, Sweden; Virginia Commonwealth University, United States of America

## Abstract

Post-cardiac arrest myocardial dysfunction is a major cause of mortality in patients receiving successful cardiopulmonary resuscitation (CPR). Mild therapeutic hypothermia (MTH) is the recommended treatment after resuscitation from cardiac arrest (CA) and is known to exert neuroprotective effects and improve short-term survival. Yet its cytoprotective mechanisms are not fully understood. In this study, our aim was to determine the possible effect of MTH on vasoactive mediators belonging to the endothelin/nitric oxide axis in our porcine model of CA and CPR. Pigs underwent either untreated CA or CA with subsequent CPR. After state-of-the-art resuscitation, the animals were either left untreated, cooled between 32–34°C after ROSC or treated with a bolus injection of S-PBN (sodium 4-[(tert-butylimino) methyl]benzene-3-sulfonate N-oxide) until 180 min after ROSC, respectively. The expression of endothelin 1 (ET-1), endothelin converting enzyme 1 (ECE-1), and endothelin A and B receptors (ETAR and ETBR) transcripts were measured using quantitative real-time PCR while protein levels for the ETAR, ETBR and nitric oxide synthases (NOS) were assessed using immunohistochemistry and Western Blot. Our results indicated that the endothelin system was not upregulated at 30, 60 and 180 min after ROSC in untreated postcardiac arrest syndrome. Post-resuscitative 3 hour-long treatments either with MTH or S-PBN stimulated ET-1, ECE-1, ETAR and ETBR as well as neuronal NOS and endothelial NOS in left ventricular cardiomyocytes. Our data suggests that the endothelin and nitric oxide pathways are activated by MTH in the heart.

## Introduction

Myocardial preservation continues to be an important determinant factor of the outcome for cardiac arrest (CA) victims after successful restoration of spontaneous circulation (ROSC). Post-resuscitation myocardial dysfunction, an important component of the “postcardiac arrest syndrome” [1]. is caused by ischemia/reperfusion (I/R) injury and includes primary manifestations such as arrhythmias, myocyte death, and contractile dysfunction so-called “stunning” [2]. In addition myocardial dysfunction aggravates persistent precipitating pathology such as chronic heart failure or angina pectoris, requiring life-long medication and clinical follow-up. To date, mild therapeutic hypothermia (MTH) is the only treatment, implemented in post-resuscitation care of after out-of-hospital cardiac arrest patients, known to improve neurological outcome and reduce mortality after CA [3], [4].

Endothelin-1 (ET-1), a 21 amino acid peptide generated by cleavage by endothelin converting enzyme-1 (ECE-1) [5], exerts its effects by binding to endothelin A (ETAR) and B (ETBR) both present in the heart [6]. ET-1 has both beneficial and detrimental roles in cardiac physiology as well as pathology [7] and is directly involved in the myocardial dysfunction following I/R injury [8].

Here we hypothesized that the ET-1 pathway could be a mediator of the action of MTH on I/R injury in the myocardium.We used a porcine model of CA and CPR reflecting a realistic simulated clinical setting and we measured expression levels of both transcripts and proteins belonging to the endothelin system e.g. ET-1, ECE-1, ETAR and ETBR as well as protein levels of endothelin system-related enzymes e.g. nitric oxide synthases (NOS) after successful ROSC in presence or in absence of MTH.

## Materials and Methods

### Ethics statement

The study's experimental protocol was approved (permit number C108/4) by the Regional Animal Review Board of Uppsala, Sweden.

### Animals

Swedish domestic piglets aged 12–14 weeks of so-called triple breed, weighing 25.8±1.3 kg were obtained from a single provider and were fasted before the experiment with free access to water. The following inclusion criteria were applied: no apparent pre-existing disease, PaCO2 between 5–5.5 kPa, PaO2>10 kPa (75 mmHg) at baseline after stabilization.

### Cardiac arrest (CA) and reperfusion models

**Figure 1:**
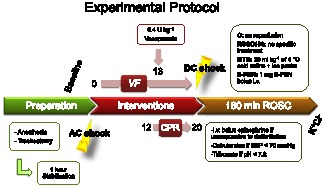
Experimental procedure. After 1 h stabilization, control pigs (C group) were sacrificed with a potassium chloride injection (KCl) after untreated cardiac arrest. Other animals were subjected to 20 min ventricular fibrillation (VF) including 12 min cardiac arrest and 8 min cardiopulmonary resuscitation (CPR) followed by return of spontaneous circulation (ROSC) and received either a saline solution (ROSC180 group), an intravenous infusion of 4°C cold saline solution (MTH group) or an intravenous infusion of sodium 4-[(tert-butylimino) methyl]benzene-3-sulfonate N-oxide (S-PBN group) before sacrifice at 180 min post-resuscitation.

Our model with 12 min untreated CA and eight min CPR has previously been described [9]. Here, we induced CA with identical anesthesia, fluid administration and surgical preparation (supporting file Preparation Protocol S1). The experimental protocol with timeline and all different interventions are summarized in Figure 1. After completion of the study, all animals received an injection of 10 mL potassium chloride 20 mmol/mL and were sacrificed. Cardiac left ventricle tissue samples were removed within 2 min after death, immediately frozen in liquid nitrogen and stored at −80°C prior to mRNA and protein analyses. The piglets were randomized into four groups: one non-resuscitated group and three resuscitated groups. The non-resuscitated group served as control (C group, n = 5) and underwent 8 min. CA. The resuscitated groups underwent 8 min CA and 12 min CPR, without subsequent hypothermia (ROSC groups, n = 18 and S-PBN group, n = 5) or with hypothermia (MTH, n = 6). The hearts were removed immediately after CA (C group) or at 30 min (ROSC30), at 60 min (ROSC60) and 180 min (ROSC180) after ROSC respectively. In the S-PBN group, a dose of 40 mg/kg sodium 4-[(tert-butylimino) methyl]benzene-3-sulfonate N-oxide (S-PBN; Sigma Aldrich) was administrated 1 min after CPR was initiated. These piglets were followed until 180 min after reperfusion. The last group underwent CA followed by CPR and hypothermia (MTH). In this group, an intravenous infusion of 4°C cold saline solution 30 ml/kg was administrated under 30 min, starting immediately after ROSC (body temperature decrease was 4.6±1.5°C) while external ice packs were used under the whole experiment time to maintain mild hypothermia (34°C). All animals received the same amount of fluid. Untreated animals were administered saline solution 30 ml/kg at room temperature. All experiments were performed in the climate-controlled animal operation room set at 21–24°C.

### RNA extraction

Total RNA was extracted with RNeasy® Fibrous Tissue Midi kit (Qiagen) according to manufacturer's instructions. One µg total RNA was reverse transcribed to cDNA with iScript cDNA Synthesis kit (Bio-Rad laboratories, USA) following manufacturer's instructions. RNA quality was assessed with Agilent 2100 Bioanalyzer (Agilent).

### Real-time quantitative polymerase chain reaction (qPCR)

Real-time qPCR was performed with MyiQ single-color detection system (Bio-Rad Laboratories, USA). Porcine ET-1, ECE-1, ETAR, ETBR and β-actin primers were described in Forni et al. [10]. Details about primers and amplicons are summarized in Table 1. The reactions were performed using IQ SYBR® Green Supermix (Bio-Rad laboratories, USA) following standard conditions recommended by the manufacturer. At the end of the amplification phase, a melting-curve analysis was carried out on the products formed. All samples were measured in triplicate. mRNA amount was normalized with β-actin. Relative amount of target genes compared to the calibrator (C) was calculated with standard curve method.

**Table 1:** Porcine primer sequences: forward (For.) and reverse (Rev.), with transcript (RT-PCR product) length and EMBL database accession number.

Primer	Sequence (5′- 3′)	Transcript length	Acc. no.*
ET-1	For.: CCTGTCTGAAGCCATCTC	109 bp	X07383
	Rev.: AGTAAGGAACGGTCTGAAC		
ETAR	For.: TCACCGTCCTCAATCTCTG	98 bp	S80652
	Rev.: CGCTGTGACCAATGGAATC		
ETBR	For.: CCCCTTCATCTCAGCAGGATT	203 bp	AY583500
	Rev.: GCACCAGCAGCATAAGCATG		
ECE-1	For.: CCATCATCAAGCACCTCCTC	108 bp	D89494
	Rev.: GCTCCTCAATCCTGGTTTCG		
β-actin	For.: ATGGTGGGTATGGGTCAGAAAG	103 bp	AF054837
	Rev.: TGGTGATGATGCCGTGCTC		

(ET-1) Endothelin-1; (ETAR) Endothelin A-receptor; (ETBR) Endothelin B-receptor; (ECE-1) Endothelin-Converting-Enzyme-1; (SDH) Succinate Dehydrogenase.

* GeneBank accession number at www.ncbi.nlm.nih.gov.

### Western blot

Proteins from cardiac left ventricle were prepared by rapid homogenization in Tissue Extraction Reagent II (Invitrogen Corporation, Carlsbad, CA) according to the manufacturer's instructions. Protein concentration was determined with the RC DC kit (Bio-Rad laboratories, USA). For each group, 50 µg protein extracts were pooled from three animals. The extracts were resolved by electrophoresis on 12% SDS-acrylamide gels and transferred to Hybond-P PVDF membranes (GE Healthcare, Uppsala, Sweden). The membranes were blocked with SuperBlock® T20 PBS (Thermo Scientific) before incubation in phosphate-buffered saline–Tween-20 with anti-ETBR (1∶200, AER-001; Alomone Labs, Jerusalem, Israel), anti-ETAR (1∶200, AER-002; Alomone Labs), anti-β-actin (1∶200, Sigma), anti-nNOS (1∶3000; Euro-Diagnostica AB, Malmö, Sweden), anti-iNOS (1∶3000; Abcam, Cambridge, MA) and anti-eNOS (1∶200; Santa Cruz Biotechnology, Santa Cruz, CA), respectively. After washing, membranes were incubated with anti-rabbit IgG-conjugated to horseradish peroxidase (1∶3000). Immunoreactive bands were visualized by enhanced chemiluminescence with Lumi-Lightplus (Roche Diagnostics). Protein band densities were digitally quantified and normalized to the loading control (β-actin) with a ChemiDoc XRS and Quantity One image analysis software (Bio-Rad laboratories, USA). At least two membranes with duplicate sample pools were analyzed.

### Immunohistochemistry (IHC)

Fixation: Cardiac left ventricle tissue was immersed in 4% buffered formalin and stored one week at 4°C before small tissue pieces (<3×5 mm) from the were cut and processed for immunohistochemistry. Following standard protocol [11], tissue pieces were dehydrated in alcohol graded series, rinsed in xylene and embedded in low-temperature paraffin (56–58°C). Multiple 3–5 µm sections were cut and collected on glass slides.

IHC of ETAR, ETBR: After deparaffinization, immunostaining was performed with polyclonal rabbit antibody (Alomone Labs, Jerusalem, Israel). ETA and ETB receptors antibodies were diluted 1∶100 and incubated over night under continuous shaking at room temperature. The sections were then incubated with biotinylated goat anti-rabbit secondary antiserum and visualized with a light microscope.

IHC of eNOS, iNOS and nNOS was performed applying the same protocol we described previously in Miclescu et al. [12].

No immunoreactivity was detected in negative controls stained with primary antibody in presence of the corresponding blocking peptide. The amount of positive cells in each group was estimated by counting staining for each group in a blinded fashion.

### Statistics

Statistics were performed with one-way analysis of variance and Tukey's Multiple Comparison Test (GraphPad Prism 5, GraphPad Software). P values p<0.05 were considered significant.

## Results

### Temperature measurements

Cold infusions were started in the MTH group at a mean pulmonary arterial (PA) temperature of 38.5 (±0.4) °C which was decreased to 34.02 (±0.04) °C. The median time after ROSC to reach the PA temperature of 34°C was 51.5±7.8 min. The hearts were removed at 180 min at a temperature of 33.5±0.1°C in the MTH group and 37.5±0.5°C in normothermic groups.

### Cardiac endogenous ET-1 and ECE-1 transcripts are induced by MTH

**Figure 2:**
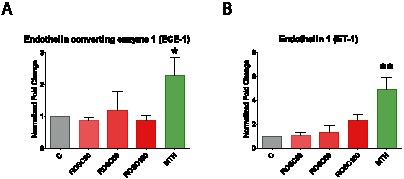
Effects of ROSC and MTH on ECE-1 and ET-1 mRNA expression analysis. Endothelin converting enzyme 1, ECE-1 (A), and endothelin 1, ET-1 (B), mRNA expression analysis was performed by real-time qPCR. The relative fold changes to control animals with untreated cardiac arrest (C group) were normalized with β-actin and calculated for animals after 30, 60 and 180 min of untreated return of spontaneous circulation (ROSC30, ROSC60 and ROSC180 groups) or after 180 min of mild therapeutic hypothermia (MTH group). Error bars represent standard error of the mean (SEM). Significant results (p<0.05) are marked with (*), (p<0.001) are marked with (***).

ET-1 is synthesized, stored and released in the human heart and is markedly involved in a number of myocardial physiological and pathophysiological processes. To study the transcriptional regulation of the endothelin system in the myocardium after ROSC we used quantitative real-time PCR. The results are expressed as the ratios of the targets normalized to the endogenous reference. In the ROSC groups where the animals went through CA followed by untreated ROSC, the expression of both ET-1 and ECE-1 mRNAs did not significantly change at 30, 60 and 180 min after resuscitation (figure 2 A and B). In the MTH group where the animals were maintained in mild hypothermia for 180 min following ROSC, both ET-1 (p<0.0001) and ECE-1 (p = 0.0127) were significantly induced (figure 2 A and B).

### ETBR transcripts are upregulated by MTH

**Figure 3:**
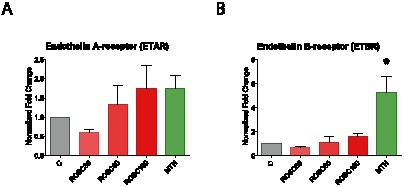
Effects of ROSC and MTH on ETAR and ETBR mRNA expression analysis. Endothelin A receptor, ETAR (A), and endothelin B receptor, ETBR (B), mRNA expression analysis was performed by real-time qPCR. The relative fold changes to control animals with untreated cardiac arrest (C group) were normalized with β-actin and calculated for animals after 30, 60 and 180 min of untreated return of spontaneous circulation (ROSC30, ROSC60 and ROSC180 groups) or after 180 min of mild therapeutic hypothermia (MTH group). Error bars represent standard error of the mean (SEM). Significant results (p<0.001) are marked with (***).

On release, ET-1 has the potential to activate specific receptors, ETAR and/or ETBR, in a paracrine manner to modify heart function directly. Consequently changes in either tissue ET-1 production or in tissue expression of its receptors can contribute to cardiac pathological states. Here we used quantitative real-time PCR to evaluate whether MTH regulates the endothelin receptors after resuscitation. The ETAR mRNA was not induced by MTH (figure 3 A) but the ETBR transcript was significantly upregulated by three hours of MTH after CPR (p = 0.0007, figure 3 B).

### The endothelin system is transcriptionally activated by S-PBN

**Figure 4:**
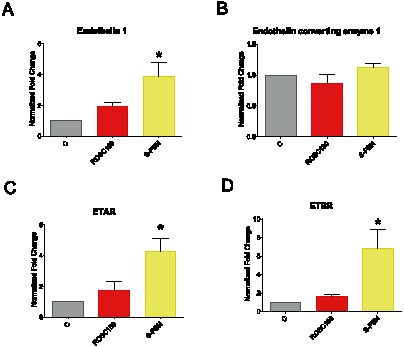
Effects of S-PBN on ET-1, ECE-1, ETAR and ETBR mRNA expression. Endothelin 1, ET-1 (A), endothelin converting enzyme 1, ECE-1 (B), endothelin A receptor, ETAR (C), and endothelin B receptor, ETBR (D), mRNA expression analysis was performed by real-time qPCR. The relative fold changes to control animals with untreated cardiac arrest (C group) were normalized with β-actin and calculated for animals after 180 min of untreated return of spontaneous circulation (ROSC180 group) or 180 min after S-PBN infusion (S-PBN group). Error bars represent standard error of the mean (SEM). Significant results (p<0.01) are marked with (**), (p<0.001) are marked with (***).

Our group is interested in identifying and testing in our swine model of CA and resuscitation new valid interventions to mitigate I/R injury following reperfusion. S-PBN (N-tert-butyl-a-phenylnitrone) is one of the candidates we previously tested on our model [13]. This compound is an organic spin trap agent designed specifically to scavenger free radicals. Beside neuroprotective effects [14], S-PBN dramatically reduces the vulnerability of the myocardium to reperfusion-induced ventricular fibrillation [15]. We hypothesized activation of the endothelin system was a common mechanism by which MTH and S-PBN exert their effect on the heart. At the transcriptional level, ET-1 (p = 0.0003), but not ECE-1, was upregulated by S-PBN after 3 hours post-ROSC (figure 4 A and B). Furthermore ETAR (p = 0.0029) and ETBR (p = 0.0057) mRNAs were both significantly induced by S-PBN treatment (figure 4 C and D).

### ETAR and ETBR are activated in cardiomyocytes by MTH and S-PBN at the protein level

**Figure 5:**
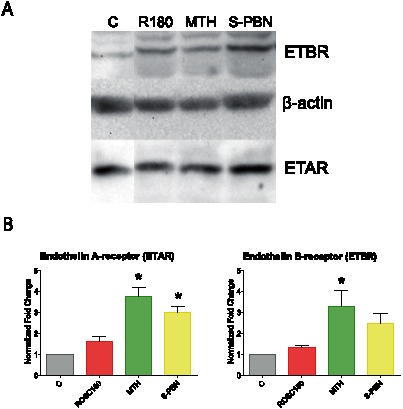
Western Blot analysis of ETAR and ETBR. Cardiac left ventricle tissue homogenates from control animals with untreated cardiac arrest (C group), or after 180 min of untreated return of spontaneous circulation (ROSC180 group), after 180 min of mild therapeutic hypothermia (MTH group) or 180 min after S-PBN infusion (S-PBN group) were loaded on a 12% SDS-acrylamide gel. Bands for endothelin A receptor (ETAR), endothelin B receptor (ETBR) and β-actin were detected at 25 kDa, 50 kDa and 42 kDa, respectively (A). Protein band intensities were quantified using a ChemiDoc XRS and Quantity One software and normalized to the loading control (β-actin) (B). Error bars represent standard error of the mean (SEM). Significant results (p<0.05) are marked with (*), (p<0.001) are marked with (***).

To verify whether MTH and S-PBN have an impact on ETAR and/or ETBR protein levels, Western Blot analyses were performed using specific antibodies for ETAR and ETBR, respectively. After digital quantification the results are presented as the ratios of ETAR and ETBR normalized with β-actin. MTH significantly upregulated both the ETAR (p<0.0001) and ETBR (p = 0.0162) proteins after resuscitation (figure 5).

**Figure 6:**
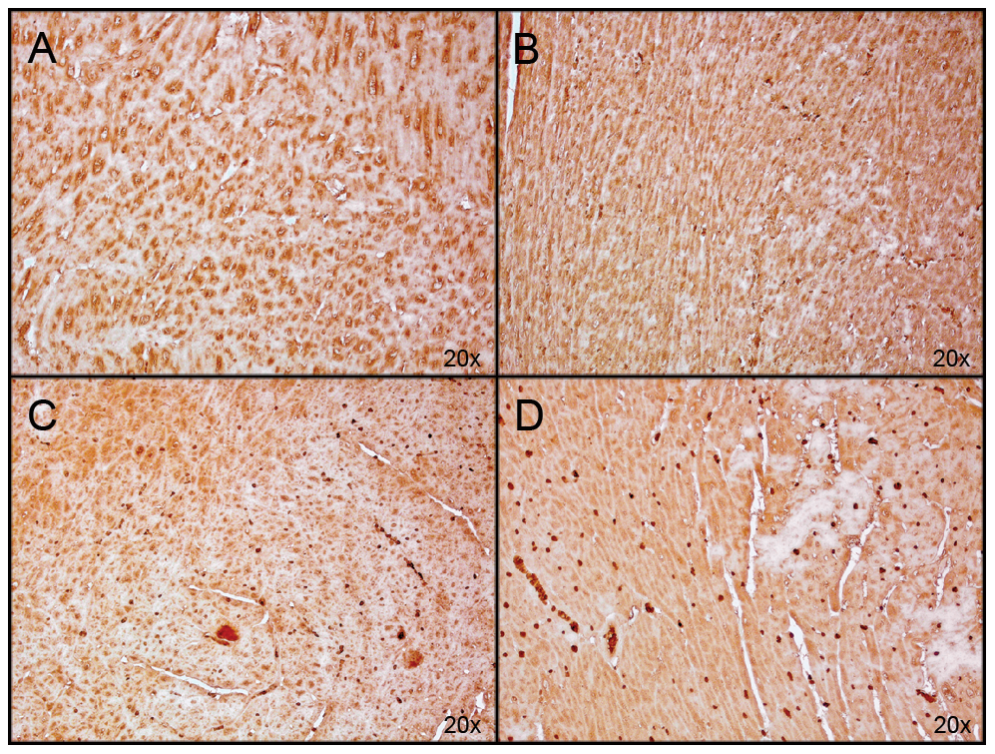
Immunohistochemistry of ETAR. Cardiac left ventricular tissue from control animals with untreated cardiac arrest (A), or after 180 min of untreated return of spontaneous circulation (B), after 180 min of mild therapeutic hypothermia (C) or 180 min after S-PBN infusion (D) were stained with anti-ETAR. The amount of ETAR-positive cardiac cells was higher in pigs treated with mild therapeutic hypothermia (C) and S-PBN (D), respectively, compared to controls (A) and untreated animals (B).

**Figure 7:**
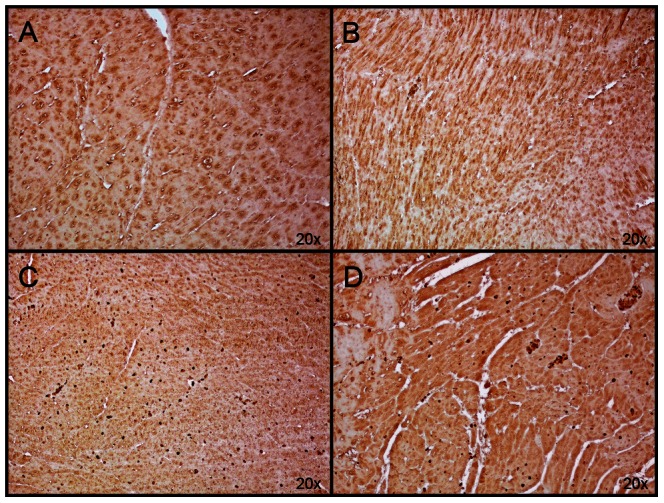
Immunohistochemistry of ETBR. Cardiac left ventricular tissue from control animals with untreated cardiac arrest (A), or after 180 min of untreated return of spontaneous circulation (B), after 180 min of mild therapeutic hypothermia (C) or 180 min after S-PBN infusion (D) were stained with anti-ETBR. The amount of ETBR-positive cardiac cells was higher in pigs treated with mild therapeutic hypothermia (C) and S-PBN (D), respectively, compared to controls (A) and untreated animals (B).

After treatment with S-PBN, ETAR was significantly increased (p<0.0001) while ETBR protein levels were elevated without reaching statistical significance (figure 5). In order for ET-1 to act in an autocrine/paracrine manner in cardiomyocytes, its receptors should be present in these cells. We detected ETAR and ETBR using immunohistochemistry in cardiac left ventricle tissue in control, untreated ROSC, MTH-treated and S-PBN treated pigs, respectively. The specificity of the anti-ETAR and anti-ETBR antibodies was verified by absence of immunoreactivity in negative controls stained with respective primary antibodies in presence of the corresponding blocking peptide (data not shown). ETAR was present in controls and untreated ROSC (figure 6 A and B) however staining of both myocytes and non-myocyte cells was stronger in MTH- and in S-PBN-treated animals (figure 6 C and D). The same pattern was observed with low expression of ETBR in controls and untreated ROSC (figure 7 A and B) and stronger staining in MTH and in S-PBN hearts (figure 7 C and D).

### Expression of NOS isoforms

Stimulation of the ET receptors induces release of endogenous nitric oxide (NO) produced by activating nitric oxide synthases. As NO plays an important role in cardioprotective effects in I/R injury, we investigated whether MTH and S-PBN treatments could influence the expression of NOS isoforms. The inducible form of NOS (iNOS) is unchanged by either MTH or S-PBN compared to ROSC180 (data not shown).

**Figure 8:**
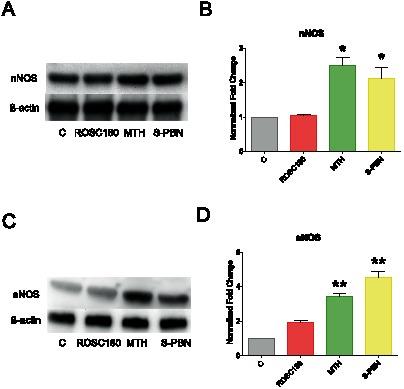
Western Blot analysis of nNOS and eNOS. Cardiac left ventricle tissue homogenates from control animals with untreated cardiac arrest (C group), or after 180 min of untreated return of spontaneous circulation (ROSC180 group), after 180 min of mild therapeutic hypothermia (MTH group) or 180 min after S-PBN infusion (S-PBN group) were loaded on a 4–20% SDS-acrylamide gel. Bands for neuronal NOS (nNOS) (A), endothelial NOS (eNOS) (C) and β-actin (A and C) were detected at 160 kDa, 135 kDa and 42 kDa, respectively. Protein band intensities for nNOS (B) and eNOS (D) were quantified using a ChemiDoc XRS and Quantity One software and normalized to the loading control (β-actin). Error bars represent standard error of the mean (SEM). Significant results (p<0.01) are marked with (**), (p<0.001) are marked with (***).

**Figure 9:**
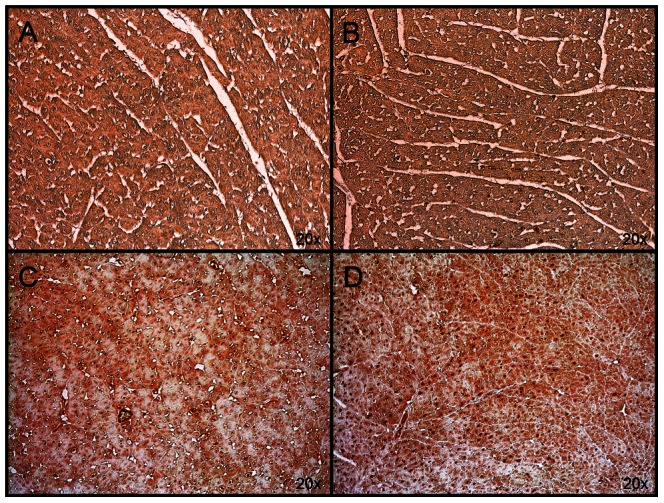
Immunohistochemistry of eNOS. Cardiac left ventricular tissue from control animals with untreated cardiac arrest (A), or after 180 min of untreated return of spontaneous circulation (B), after 180 min of mild therapeutic hypothermia (C) or 180 min after S-PBN infusion (D) were stained with anti-eNOS. The amount of eNOS-positive cardiac cells was higher in animals treated with mild therapeutic hypothermia (C) and S-PBN (D), respectively compared to controls (A) and untreated animals (B).

**Figure 10:**
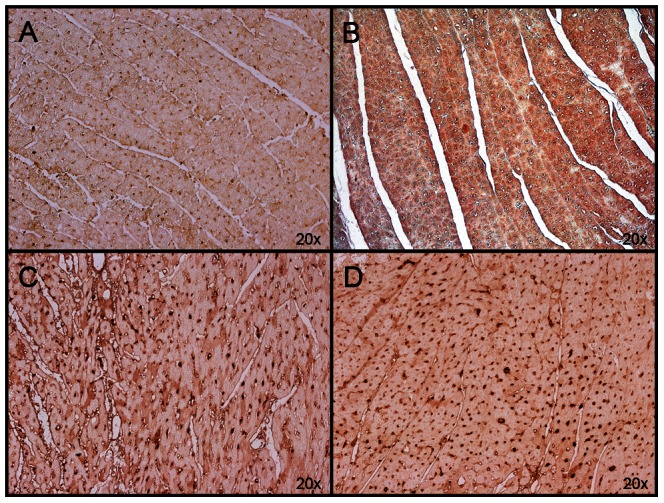
Immunohistochemistry of nNOS. Cardiac left ventricular tissue from control animals with untreated cardiac arrest (A), or after 180 min of untreated return of spontaneous circulation (B), after 180 min of mild therapeutic hypothermia (C) or 180 min after S-PBN infusion (D) were stained with anti-nNOS. The amount of nNOS-positive cardiac cells was higher in animals treated with mild therapeutic hypothermia (C) and S-PBN (D), respectively compared to controls (A) and untreated animals (B).

Western Blot results show that neuronal NOS (nNOS) was significantly upregulated by MTH and S-PBN (p = 0.0018) (figure 8 A and B). The other constitutive form of NOS, endothelial NOS (eNOS) was activated by both MTH and S-PBN (p<0.0001) (figure 8 C and D). Immunohistochemical analysis confirmed the Western Blot results and demonstrates that eNOS (figure 9) and nNOS were stimulated in cardiomyocytes (figure 10).

## Discussion

In the present study we demonstrated for the first time in a model of global ischemia–reperfusion injury after cardiac arrest that MTH induces the endothelin system . Not only myocardial endogenous ET-1 but also ECE-1, ETAR and ETBR were upregulated at both mRNA and protein levels. Concomitantly we observed activation by MTH of eNOS and nNOS three hours after ROSC. Furthermore we showed that similar post-ROSC stimulation of the endothelin system and of eNOS and nNOS occurred in animals treated with another protective agent, S-PBN. We did not find any significant changes from 30 min to 3 hours after ROSC in myocardial ET-1 or ET receptors expressions neither at mRNA nor at protein levels. Considering that it has been demonstrated that ET-1 is upregulated in porcine cardiomyocytes subjected to ischemia [16] and that it is widely documented that the endothelin system is an important factor in determining the outcome of myocardial ischemia and reperfusion (see for review [17]), our results were somewhat surprising. However the majority of these observations were made in the context of myocardial infarction and/or cardiovascular diseases therefore their conclusions might not apply to our model. Indeed two previous clinical studies, both in agreement with our results, suggest that endothelin levels are not elevated following resuscitation [18], [19]. The role of the endothelin system in CA and ROSC has not been well characterized and to our knowledge there are no reports in literature about the effect of post-resuscitative interventions on myocardial endothelin production. Here we show for the first time that MTH stimulates the endothelin system in the heart three hours after resuscitation. The mechanism by which MTH exerts its effect is thought to be multifactorial as showed by a recent study demonstrating that MTH acts by modulating inflammation, apoptosis and remodeling after CPR [20]. Few studies have addressed the potential role that ET-1 may have in CA and ROSC. There is evidence from a comparative clinical study between survivors and non-survivors that survival after CPR is associated with higher plasma endothelin concentration [18]. In addition, it was recently reported that local expression of ET-1 plays an important role in preserving cardiac myocytes exposed to stress and is a potent survival factor that protects cells from apoptosis in cardiomyocytes [21]. Our results showed that the endothelin system is also stimulated by S-PBN, an antioxidant with reactive oxygen species (ROS) scavenging properties, which has been studied by our group as a potential postresuscitative therapy [13]. In addition to its neuroprotective properties, S-PBN has powerful anti-arrhythmic and cardioprotective functional activities during I/R [22]. Our observations in MTH and S-PBN taken together suggest that stimulation of the endothelin system might be involved in the positive effect of these post-ROSC interventions. ET-1 exerts complex cardiac effects (including modulation of contractility) which are mediated by two receptors ETAR and ETBR, both found in cardiac myocytes. The ETAR is more abundant (90%) and has been considered more important for the cardiac effects of ET-1 but the ETBR may be more responsive to physiological stress [7]. In our model both the ETAR and ETBR are similarly increased by MTH and S-PBN. Defining the relative roles of ETAR versus ETBR activation in the mediation of responses to ET-1 is complicated due to a phenomenon of inhibition/compensation e.g. cross-talk between the ETAR and ETBR [23]. Therefore it is unclear which receptor might be involved in the effects of MTH and S-PBN. Whereas it has been shown that ET-1 exerts arrhythmogenic effects mainly via stimulation of the ETAR [24], a recent report demonstrated that the ETBR has a protective role on post ischemic myocardial dysfunction [25]. And while the pathways downstream of the ETBR are multiple, NO is thought to be a key molecule in its receptor-mediated actions. Indeed NO produced by ETBR activation is an important factor for the cardioprotective effects by exogenous ET-1 [26]. Therefore we further analyzed the effects of MTH and S-PBN on three different isoforms of NOS, neuronal NOS (nNOS), inducible NOS (iNOS) and endothelial NOS (eNOS). Our results revealed that both MTH and S-PBN activate eNOS and nNOS but not iNOS. This activation of eNOS and nNOS generates an increase of NO production in the myocardium and suggests that NO may be involved in the effect of MTH in the heart after CPR. However, it is unclear to what extent eNOS and nNOS contribute to the effects of MTH. The beneficial effect of eNOS on myocardial dysfunction after I/R is well characterized in the ischemic heart in the context of cardiovascular diseases (see for review [27]). However to our knowledge there is only one report in a mouse model of CA which demonstrated that genetic deletion of eNOS decreases ROSC rate and worsens post-ROSC left-ventricular function [28]. Meanwhile the physiological role of nNOS in the ischemic myocardium is slowly emerging with some evidence suggesting that nNOS may be considered chiefly responsible for physiological NO-mediated autocrine regulation of cardiomyocyte contraction and relaxation, mainly through modulation of excitation-contraction [29]. In addition a recent study indicated that overexpression of nNOS results in myocardial protection after I/R injury [30]. In the heart nNOS has been suggested to be the isozyme targeted to the mitochondria where it generates NO within the organelle. Through its interaction with components of the electron-transport chain, NO functions as a physiological regulator of cell respiration and production of reactive species. Modulation of nNOS activity by MTH could therefore result in regulating mitochondrial NO production and optimize the balance between cardiac energy production and utilization and to regulate processes such as apoptosis, oxygen and nitrogen free radical production and Ca^2+^ homeostasis.

Future studies with specific inhibitors of NOS isoforms are required to better understand the respective roles of eNOS and nNOS in the effect of MTH and S-PBN in the myocardium. Likewise additional experiments with inhibitors specific to ETAR and ETBR are necessary to determine the relationship between both forms of ET receptors and their actions on NOS activation.

MTH is an established intervention which has shown benefits in patients who have suffered cardiac arrest however the survival to hospital discharge rate for victims after successful ROSC is still disappointing [31] leaving a large unmet medical need. The physiological effects of MTH are multifaceted and therefore a better knowledge of its underlying molecular mechanisms is essential for the development of innovative combination therapies to augment the protective benefits of hypothermia. We showed that the ET/NOS pathway is a therapeutic target of MTH. Therefore drugs modulating this pathway might be combined with MTH to either potentially enhance its overall protection or prolong its temporal therapeutic window.

## Conclusion

We demonstrated for the first time that two postresuscitative interventions, MTH and S-PBN, activate ET-1 and its receptors concomitantly with eNOS and nNOS in the myocardium. We concluded that NO and ET pathways are activated by MTH and could be considered as valuable therapeutic targets for novel post-resuscitative drug discovery.

## Supporting Information

Preparation Protocol S1 Animal preparation and procedures for induction of cardiac arrest and cardiopulmonary resuscitation.
(PDF)
