# Supplementary material for: Therapeutic Hypothermia Activates the Endothelin and Nitric Oxide Systems after Cardiac Arrest in a Pig Model of Cardiopulmonary Resuscitation
Source: PLoS One. 2013 May 23;8(5):e64792. doi: 10.1371/journal.pone.0064792 (PMC3662665; doi:10.1371/journal.pone.0064792)
Supplement: Preparation Protocol S1 — Animal preparation and procedures for induction of cardiac arrest and cardiopulmonary resuscitation. (PDF) [file pone.0064792.s001.pdf]

Preparation Protocol S1

Anesthesia and fluid replacement

To induce general anesthesia, 6 mg kg−1 tiletamine-zolazepam (Reading, France), 2.2 mg kg−1 xylazine (Bayer, Germany) and 0.04 mg kg−1 atropine was injected intramuscularly. Animals were placed on a warming blanket to maintain baseline temperature (37.5–38.5 °C) during preparation for the experiment in all groups, and during the experiment in the normothermic groups. All experiments were performed in the same climate-controlled animal surgical suite set at 21–24°C. After the first boluses of ketamine 2 mg/kg and morphine 0.1 mg/kg, an intravenous infusion of 8 mg/kg/h sodium pentobarbital, morphine 0.5 mg/kg/h and 0.25 mg/kg/h pancuronium bromide was started for maintenance of anesthesia and shivering prevention. Adequate anesthesia was confirmed by absence of motor and sympathetic responses to painful stimuli. The piglets were tracheotomized and mechanically ventilated (Servo 900C, Siemens-Elema, Solna, Sweden) with 30% oxygen/air mix, 1:2 inspiratory:expiratory (I:E) ratio, 25 min−1 respiratory frequency, 5 cm H2O positive end-expiratory pressure (PEEP) and minute volume set to maintain arterial partial pressure of carbon dioxide (PaCO2) between 5.0 and 5.5 kPa. The capnogram, peripheral oxygen saturation and electrocardiogram (ECG) were displayed continuously until the end of the experiment. Temperature probes were inserted for measuring esophageal (Tesof) and rectal temperature (Tr). During the first hour of preparation, fluid replacement with acetated Acetated Ringer´s solution was standardized for all animals with 30 mL kg^{-1}, followed by continuous infusion of 10 mL kg^{-1} h^{-1} acetated Ringer´s solution and 8 mL kg^{-1} h^{-1} 2.5% glucose-electrolyte solution.

Surgical preparation

An 18-G arterial catheter was advanced into the aortic arch via a branch of the right subclavian artery for withdrawal of blood samples and measurement of blood pressure. A 14-G saline-filled double lumen catheter was placed into the right atrium via a cutdown of the right external jugular vein. Along with that, a pulmonary artery catheter was inserted to monitor the temperature, cardiac output (CO), pulmonary artery pressure (PAP) and capillary wedge pressure (PCWP). The bladder was catheterized to collect urine and monitor diuresis.

Measurements and samples

Peripheral oxygen saturation and capnograms (CO_2SMO Plus-8100, Novametrix, Wallingford, CT, USA) as well as hemodynamic parameters, including heart rate, systemic arterial blood pressure, electrocardiogram leads II and V5, right atrial pressure and pulmonary artery pressure were monitored and recorded continuously (BioPac MP100, Acknowledge, version 3.8.1 software, BioPac Systems, Santa Barbara, CA). Cardiac output was measured with thermodilution technique and coronary perfusion pressure (CPP) was calculated. The CO, PCWP (measured at baseline and at 30, 60, 120, 180 min after return of spontaneous circulation (ROSC)) and CPP were recorded and measured according to the previous protocols (Clark CA, Harman EM: Hemodynamic Monitoring: Pulmonary Artery Catheters. In Critical Care, 3rd edition. Civetta JM, Taylor RW, Kirby RR. Philadelphia: Lippincott Williams & Wilkins 1988, pp293-302). Samples of arterial blood were taken for blood gas analysis and acid-base balance at regular intervals. Oxygen saturation and hemoglobin were determined, at the same time points.

Experimental protocol

Baseline measurements were taken after 1 h stabilization and ventilation with 0.3/0.7 oxygen/air mix. To induce ventricular fibrillation, a 50-Hz, 20-60 V alternating transthoracic current was applied via two subcutaneous needle electrodes. Cardiopulmonary arrest was defined as ventricular fibrillation in the electrocardiogram combined with aortic blood pressure below 25 mm Hg. In the control group, experimentation was ended without treatment directly after cardiac arrest. The other three groups underwent twelve minutes untreated cardiac arrest without mechanical ventilation. Thereafter, closed-chest cardiopulmonary resuscitation (CPR) was performed by an automatic device (Lucas™, Jolife AB, Lund, Sweden) and mechanical ventilation was resumed with 100% oxygen and otherwise unchanged parameters. Following one minute CPR, all animals received a bolus 0.4 U kg^{-1} vasopressin (Arg^8-vasopressin, PolyPeptide Laboratories, Wolfenbüttel, Germany) via the right atrial catheter. After eight minutes of external chest compressions, a 200 J monophasic counter shock was delivered through defibrillation electrode pads (Medtronic Physio-Control Corp., Seattle, WA, USA). If spontaneous circulation was not restored, CPR continued 2 min followed by defibrillation (200J). A bolus of epinephrine (20 μg kg^{-1}) was administered after the third DC shock. CPR and DC shocks were applied over maximally 5 min according to resuscitation guidelines from 2010 (Nolan JP, Soar J, David A. Zideman DA, et al: European Resuscitation Council Guidelines for Resuscitation 2010. Section 1. Executive summary. Resuscitation. 2010; 81: 1219–1276). If ROSC was not achieved within this timeframe, CPR was discontinued. ROSC was defined as return of coordinated electrical activity resulting in systolic blood pressure > 60 mm Hg for at least ten minutes. Oxygen was reduced to 30% after 5 min of spontaneous circulation. Acidosis was corrected by increasing minute ventilation and with 1 mmol kg^{-1} tris buffer mixture (Tribonat®, Kabi Fresenius, Stockholm, Sweden) on acidosis, defined as arterial pH <7.20 or base deficit >10 mmol L^{-1}, 5 min after ROSC. Dobutamine was infused to maintain systolic blood pressure >70 mm Hg in a dose of

5 mcg/kg/h. Thereafter, the animals were treated according to the protocols described in the materials and methods section.
